# Limited Effects of Inorganic Nitrate Supplementation on Exercise Training Responses: A Systematic Review and Meta-analysis

**DOI:** 10.1186/s40798-023-00632-1

**Published:** 2023-09-11

**Authors:** Austin C. Hogwood, Kara C. Anderson, Joaquin Ortiz de Zevallos, Craig Paterson, Arthur Weltman, Jason D. Allen

**Affiliations:** 1 Department of Kinesiology, School of Education and Human Development, University of Virginia, Charlottesville, VA 22904 USA; 2 Department of Exercise and Sport Science, University of North Carolina, Chapel Hill, NC 27599 USA; 3 Department of Medicine, University of Virginia School of Medicine, Charlottesville, VA 22904 USA

**Keywords:** Inorganic nitrate, Exercise training, VO_2peak_, Time to exhaustion

## Abstract

**Background:**

Inorganic nitrate (NO_3_^−^) supplementation is purported to benefit short-term exercise performance, but it is unclear whether NO_3_^−^ improves longer-term exercise training responses (such as improvements in VO_2peak_ or time to exhaustion (TTE)) versus exercise training alone. The purpose of this systematic review and meta-analysis was to determine the effects of NO_3_^−^ supplementation combined with exercise training on VO_2peak_ and TTE, and to identify potential factors that may impact outcomes.

**Methods:**

Electronic databases (PubMed, Medscape, and Web of Science) were searched for articles published through June 2022 with article inclusion determined *a priori* as: (1) randomized placebo-controlled trials, (2) exercise training lasted at least three weeks, (3) treatment groups received identical exercise training, (4) treatment groups had matched VO_2peak_ at baseline. Study quality was assessed using the Cochrane Risk-of-Bias 2 tool. Standardized mean difference (SMD) with 95% confidence intervals (CI) were calculated using restricted maximum likelihood estimation between pre- and post-training differences in outcomes. Moderator subgroup and meta-regression analyses were completed to determine whether the overall effect was influenced by age, sex, NO_3_^−^ dosage, baseline VO_2peak_, health status, NO_3_^−^ administration route, and training conditions.

**Results:**

Nine studies consisting of eleven trials were included: n = 228 (72 females); age = 37.7 ± 21 years; VO_2peak_: 40 ± 18 ml/kg/min. NO_3_^−^ supplementation did not enhance exercise training with respect to VO_2peak_ (SMD: 0.18; 95% CI: -0.09, 0.44; *p* = 0.19) or TTE (SMD: 0.08; 95% CI: − 0.21, 0.37; *p* = 0.58). No significant moderators were revealed on either outcome. Subset analysis on healthy participants who consumed beetroot juice (BRJ) revealed stronger trends for NO_3_^−^ improving VO_2peak_ (*p* = 0.08) compared with TTE (*p* = 0.19), with no significant moderators. Sunset funnel plot revealed low statistical power in all trials.

**Conclusions:**

NO_3_^−^ supplementation combined with exercise training may not enhance exercise outcomes such as VO_2peak_ or TTE. A trend for greater improvement in VO_2peak_ in healthy participants supplemented with BRJ may exist (*p* = 0.08). Overall, future studies in this area need increased sample sizes, more unified methodologies, longer training interventions, and examination of sex as a biological variable to strengthen conclusions.

**Supplementary Information:**

The online version contains supplementary material available at 10.1186/s40798-023-00632-1.

## Key Points

- Despite the increased use and study of NO_3_^−^ supplementation, most of these data have shown benefits during acute supplementation on exercise rather than a benefit to chronic training outcomes.
- This systematic review and meta-analysis revealed non-significant improvements in VO_2peak_ or time to exhaustion after exercise training with NO_3_^-^ supplementation compared to exercise alone. A trend for improvement was found for improvements in healthy participants taking beetroot juice supplementation.
- These results suggest that NO_3_^-^ may not have an impact on improving longer term training outcomes, but studies in this area suffer from low sample sizes and inconsistent study designs.

**Supplementary Information:**

The online version contains supplementary material available at 10.1186/s40798-023-00632-1.

## Background

Inorganic nitrate (NO_3_^−^) is found in high abundance in various green leafy vegetables and roots. Although NO_3_^−^ itself is thought to be relatively inert, it is converted via entero-salivary bacterial reduction in the oral cavity and gut to nitrite (NO_2_^−^) [1, 2]. Subsequently, NO_2_^−^ can be converted to nitric oxide (NO) in conditions of low oxygen tension and mildly acidic pH in a process facilitated by deoxyhemoglobin and enzymes such as xanthine oxidase [3]. Therefore, exercise at a relatively high-intensity results in conditions favorable for increased reduction of NO_2_^−^ to NO. NO has been shown to play a potentially beneficial role in several physiological processes linked to exercise performance, including increased blood flow and O_2_ delivery [4, 5], increased microvascular PO_2_ [6], improved muscular contractility [7, 8], and reduced O_2_ cost of exercise [9–11] by enhancing mitochondrial function [12]. This suggests a potential for enhanced exercise training benefits, as NO may increase acute exercise responses which may in turn accumulate into an increased training response. Contrarily, these acute findings are equivocal, and it is possible that factors such as the decreased exercise-induced muscle perturbations found after NO_3_^−^ supplementation [13] may result in lessened adaptation in response to exercise training.

The role of exogenous NO_3_^−^ supplementation in human exercise performance has generated a great deal of academic interest in the last decade [14]. Several high-quality meta-analyses have been performed, and most recently an expert consensus paper using the modified Delphi technique concluded that acute and chronic NO_3_^−^ supplementation was likely safe up to 16 mmol/day when consumed over several weeks, and that it is likely to produce ergogenic benefits during acute exercise in individuals with lower and more moderate aerobic fitness (i.e., those with VO_2peak_ > 60 ml/kg/min have shown generally less benefit) [15]. Despite this, whether NO_3_^−^ supplementation causes acute improvements in factors such as exercise endurance in each exercise training session, subsequently resulting in an accumulated larger training volume and greater adaptation for outcomes such as VO_2peak_, remains unclear.

VO_2peak_ is considered the criterion measure of cardiorespiratory adaptation to exercise training [16, 17]. VO_2peak_ improves with exercise training via multifaceted improvements in oxygen delivery (e.g., stroke volume, blood volume) and oxygen utilization (e.g., a-vO_2_ difference), and is a predictor of both endurance capacity and mortality/morbidity [18]. Although VO_2peak_ primarily quantifies aerobic fitness, there is considerable variability in the endurance capacity of individuals with similar VO_2peak_ [19]. More practical measures such as exercise time-to-exhaustion (TTE) may better represent competitive endurance performance (as this is likely to be more closely related to the percent of VO_2peak_ associated with the lactate threshold and/or the lactate turn point) and offer additional insight into exercise training adaptations. As such, many exercise training studies evaluate changes to both VO_2peak_ and TTE in a relatively consistent manner, making comparison across studies possible.

The purpose of this systematic review and meta-analysis is to explore whether NO_3_^−^ supplementation can provide additional benefits when combined with chronic exercise training, and to determine whether factors such as baseline fitness, sex, health status, NO_3_^−^ dosage, route of NO_3_^−^ administration, and training conditions may moderate the effects of NO_3_^−^ on training outcomes.

## Methods

This systematic review and meta-analysis was performed in accordance with PRISMA (Preferred Reporting Items for Systematic Reviews and Meta-Analyses) guidelines [20] but was not pre-registered. Covidence (Covidence systematic review software, Veritas Health Innovation, Melbourne, Australia) was used for title, abstract, and full-text screening.

### Literature Search

Electronic databases (PubMed, Medscape, and Web of Science) were searched by two authors in tandem, ACH and JOZ, with articles published from inception of the databases through June 2022 included. The search used the following terms: (((((("Dietary nitrate") OR ("Inorganic nitrate")) AND (Training)) NOT (Acute)) NOT (Rat)) NOT (rodent)) NOT (mouse)). Reference lists of all relevant studies along with reviews and book chapters were also examined. Articles were limited to randomized controlled trials (RCTs) in the English language.

### Article Selection

For this meta-analysis, the term ‘article’ is used synonymously with ‘study’, and ‘trial’ is the unit included in the meta-analysis. Articles sometimes contained multiple eligible trials that comprised an intervention group and a comparable control.

First, the titles and abstracts of the articles were screened for eligibility. The following criteria were determined a priori for article inclusion: (1) the study was a RCT, (2) exercise training (i.e., repeated bouts of exercise multiple times per week) lasted at least 3 weeks, (3) the placebo and NO_3_^−^ group received identical exercise training, (4) the placebo and NO_3_^−^ had matched VO_2peak_ at baseline. Full texts were reviewed of the remaining articles to determine eligibility. Two authors (ACH and JOZ) independently completed the study selection.

### Data Extraction and Bias Assessment

Articles meeting inclusion criteria had the following data extracted and systematically organized: (1) author and publication year; (2) continuous variables: change in VO_2peak_, change in time to exhaustion, sample size, baseline VO_2peak_, age, body weight (kg), duration of training intervention, and NO_3_^−^ dosage both in mmol and in mmol/kg/day; (3) categorical variables: sex, route of NO_3_^−^ administration, environmental oxygenation during training, training intensity, health status, training modality, and whether an acute dose of NO_3_^−^ was taken prior to the final post-training testing. If NO_3_^−^ mmol/kg/day data were not provided, they were manually calculated based on mmol and mean kg data. If data were not available in the articles, authors were contacted for data.

Study quality was assessed using the Cochrane risk-of-bias tool for randomized trials (RoB 2) for both outcomes which includes the following domains: randomization, deviations from interventions, missing outcome data, measurement of outcome data, and results [21]. In each domain are signaling questions, where the risk of bias calculated from each domain is generated from an algorithm. Each study is scored as either “low risk”, “high risk” or “some concern” of bias based on the answers to the signaling questions. Two authors (ACH and JOZ) independently answered the signaling questions. Additionally, the Grading of Recommendations Assessment, Development and Evaluation (GRADE) using GRADEPro was performed to assess the certainty of evidence for the outcomes and sensitivity analyses. Certainty of evidence for each outcome was assessed using the following scale: “high,” “moderate,” “low,” or “very low” certainty. Certainty of each outcome was downgraded due to (1) risk of bias/study limitations, (2) inconsistency in results, (3) indirectness of results, (4) imprecision, or (5) reporting/publication bias. Two authors (ACH and CP) independently assessed the certainty of evidence.

### Statistical Analysis

The meta-analysis and subgroup analysis was performed using Open Meta-Analyst, whereas funnel plots, Cook’s distance, studentized residuals, and sunset plots [22] were calculated and plotted using ‘metafor’ [23] in RStudio (Version 1.3.1073). Data were considered statistically significant *a priori* at *p* < 0.05 and data are presented as standardized mean difference (SMD).

A random effects model with restricted maximum likelihood estimation was utilized. The SMD of both VO_2peak_ and TTE between the NO_3_^−^ groups and the placebo groups of each trial were utilized in the model to determine the pooled effect. SMD was utilized due to inconsistent reporting of VO_2peak_ in absolute or relative units. The SMDs are expressed as Hedges' *g* to account for any bias due to small sample sizes within trials. The Hedges' *g* values are interpreted as follows: ≤ 0.2, 0.2, 0.5, and 0.8 are considered to represent trivial, small, moderate, and large effect sizes, respectively [24].

The robustness of the pooled results was examined via funnel plots for small study effects. Sensitivity analysis was performed using Cook's distance and studentized residuals to identify potentially influential or outlying trials, respectively [25]. If a study was identified as being potentially influential or outlying, the robustness of the overall analysis was tested by removing the identified trial(s). A second sensitivity analysis was performed on only studies administering NO_3_^−^ in the form of beetroot juice (BRJ) in healthy participants only. Statistical heterogeneity of the overall model was assessed with using Cochrane’s Q and *I*^2^, where < 25% indicates low risk of heterogeneity, 25–75% indicates moderate risk of heterogeneity, and > 75% indicates considerable risk of heterogeneity [26]. A sunset funnel plot was used to visualize the statistical power of each trial [27].

Moderator analysis was performed to determine whether the following subgroups impacted pooled effects: sex, health status, route of NO_3_^−^ administration, and whether participants trained under normoxic or hypoxic conditions. While training intensity and modality were of interest, the included trials that had healthy populations all trained both at a high intensity while cycling, whereas all trials included that had clinical populations trained at a moderate intensity on a treadmill. Because of this, subgroup analysis for health status was the same subgroup for exercise intensity and modality. Meta-regressions were also performed to determine whether the following continuous variables impacted pool effects: age, weight, baseline VO_2peak_, NO_3_^−^ dosage in mmol per day, and NO_3_^−^ dosage in mmol/kg/day. The regression coefficient (ß) is reported along with 95% confidence intervals (CI).

## Results

### Literature Search

**Fig. 1:**
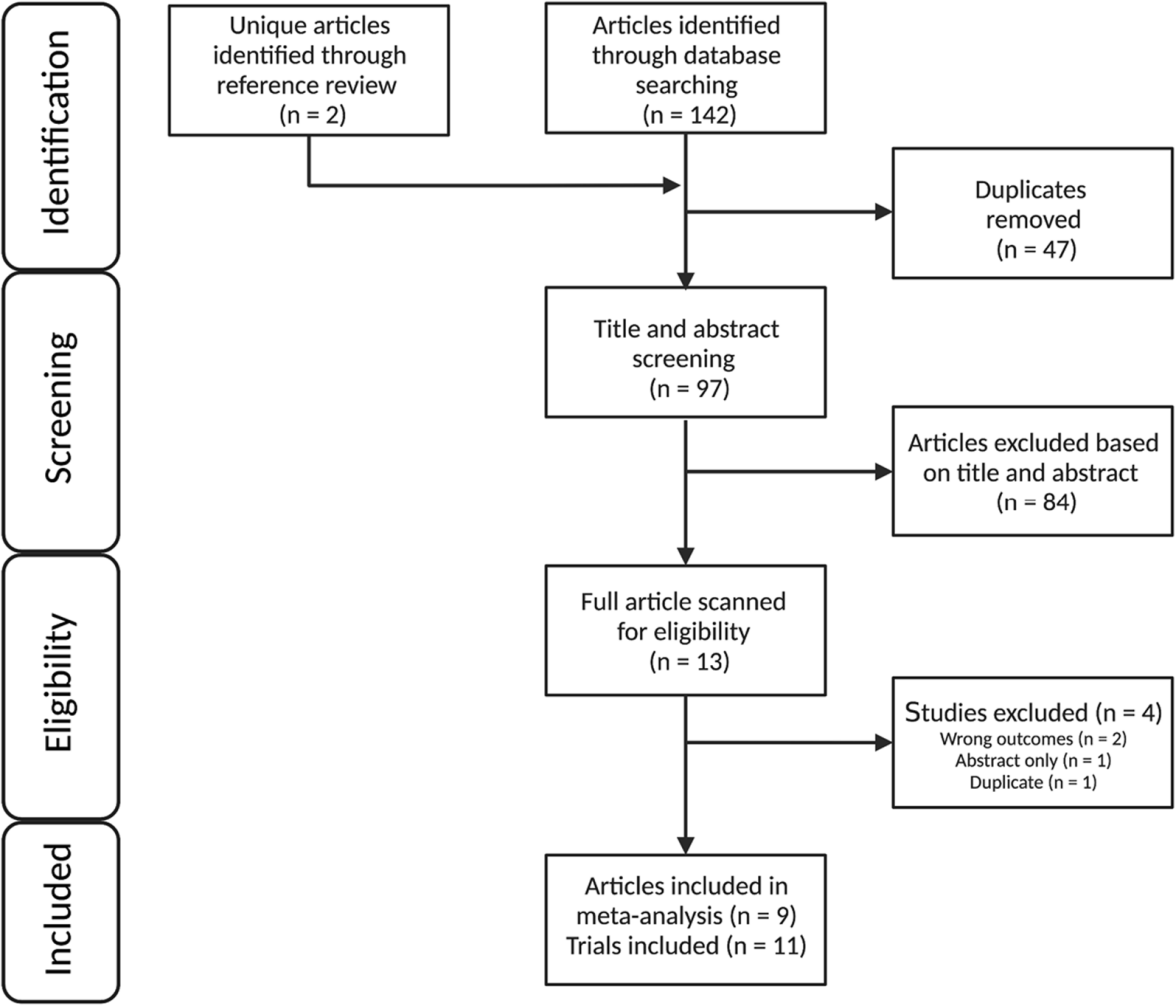
Flowchart of article and trial selection

The PRISMA flow diagram outlining the literature search is presented in Fig. 1. In total, 144 references were imported for screening, with 47 duplicates removed leaving 97 studies screened against title and abstract. Following screening of titles and abstracts (ACH and JOZ), 84 studies were excluded leaving 13 studies assessed for full-text eligibility. Of these, 4 studies were excluded, leaving 9 studies [28–36] which met inclusion criteria containing 11 eligible trials. The trial participant and supplement characteristics are shown in Table 1, while the trial exercise training characteristics are shown in Table 2.

**Table 1:** Trial participant and supplementation characteristics

Trial	Total sample size	Sex	Age (yrs)	Weight (kg)	Baseline VO_2peak_ (ml/kg/min)	NO_3_^−^ dose (mmol/day)	NO_3_^−^ dose (mmol/kg/day)*	Acute dose prior	Health status	Route of administration
De Smet et al. [28]	17	M	24.5	79.0	52.8	6.5	0.082	No	Healthy	BRJ
Finkel et al. [29]	16	M	26.7	77.9	56.7	–	0.140	No	Healthy	Other
Muggeridge et al. [30]	19	M	28.5	82.3	41.0	8.0	0.097	No	Healthy	Other
Puype et al. [31]	22	M	21.6	72.2	60.5	11.3	0.070	Yes	Healthy	BRJ
Shaltout et al. [32a]	19	M/F	69.3	87.8	11.9	6.1	0.069	Yes	Clinical	BRJ
Shaltout et al. [32b]	27	M/F	65.3	97.8	18.4	8.0	0.082	Yes	Clinical	BRJ
Sousa et al. [33]	20	M	36.8	70.6	56.2	8.4	0.120	Yes	Healthy	BRJ
Thompson et al. [34]	24	M/F	25.0	72.5	45.1	12.8	0.180	Yes	Healthy	BRJ
Thompson et al. [35a]	24	M/F	23.5	75.5	43.3	12.8	0.170	Yes	Healthy	BRJ
Thompson et al. [35b]	24	M/F	23.5	75.5	43.3	12.8	0.170	Yes	Healthy	Other
Woessner et al. [36]	24	M/F	69.7	80.5	14.7	4.2	0.050	No	Clinical	BRJ

Data are presented as means

*M* males, *F* females, *Yrs* years, *Kg* kilograms; [32a] Heart failure trial. [32b] Hypertension trial. [35a] Beetroot juice trial. [35b] Potassium nitrate trial

*Manually calculated based on mmol and mean kg data

**Table 2:** Trial exercise training characteristics

Trial	Classification	Training duration (weeks)	Training time (min)	Frequency (sessions per week)	Modality	Oxygen status
De Smet et al. [28]	SIT	5	30–40	3	Cycling	H
Finkel et al. [29]	HIHVT	3	45	3	Cycling	N
Muggeridge et al. [30]	HIIT	3	17	3	Cycling	N
Puype et al. [31]	HIET	6	30	5	Cycling	H
Shaltout et al. [32a]	Moderate/Vigorous	4	40	3	Treadmill/Cycling	N
Shaltout et al. [32b]	Mod	6	50	3	Treadmill	N
Sousa et al. [33]	HIIT/SIT	4	52–57	3	Cycling	H
Thompson et al. [34]	SIT	4	18–22.5	3–4	Cycling	N
Thompson et al. [35a]	SIT	4	18–22.5	3–4	Cycling	N
Thompson et al. [35b]	SIT	4	18–22.5	3–4	Cycling	N
Woessner et al. [36]	HIIT/Rehab	12	30	3–4	Treadmill	N

Data are presented as means

*SIT* Sprint interval training, *HIHVT* High intensity high volume training, *HIIT* High intensity interval training, *HIET* High intensity endurance training

Oxygen Status; *H* Hypoxia, *N* Normoxia. [32a] Heart failure trial. [32b] Hypertension trial. [35a] Beetroot juice trial. [35b] Potassium nitrate trial

### Risk of Bias

Both outcomes produced similar results on the RoB analysis. Of the 9 included studies, 7 were considered to have a low risk of bias, with 2 having some concern due to the nature of their single-blinded supplement design [33] or lack of explicit mentioning of double-blinding [31] (Additional file 1: Figure S1).

### Pooled Effects

**Fig. 2:**
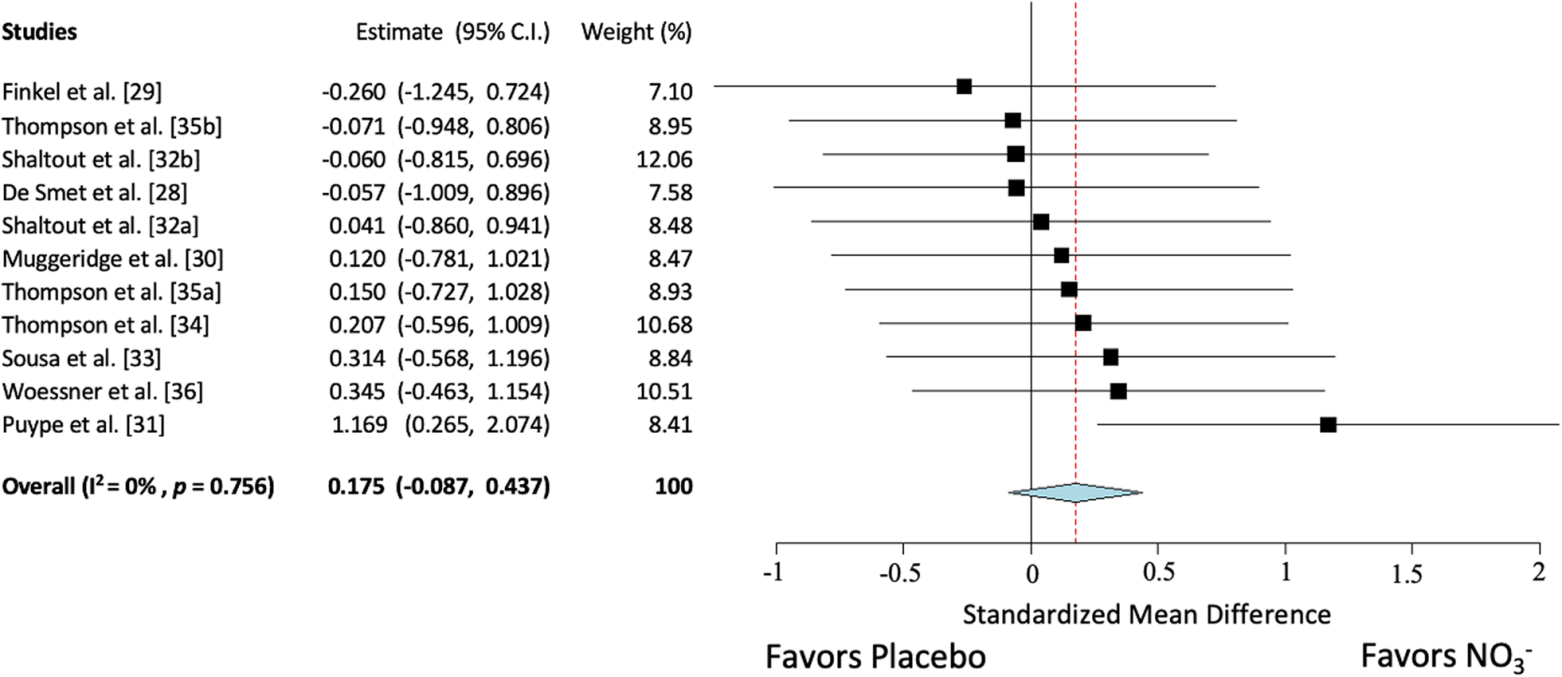
Forest plot of VO_2peak_. [32a] Heart failure trial. [32b] Hypertension trial. [35a] Beetroot juice trial. [35b] Potassium nitrate trial

**Fig. 3:**
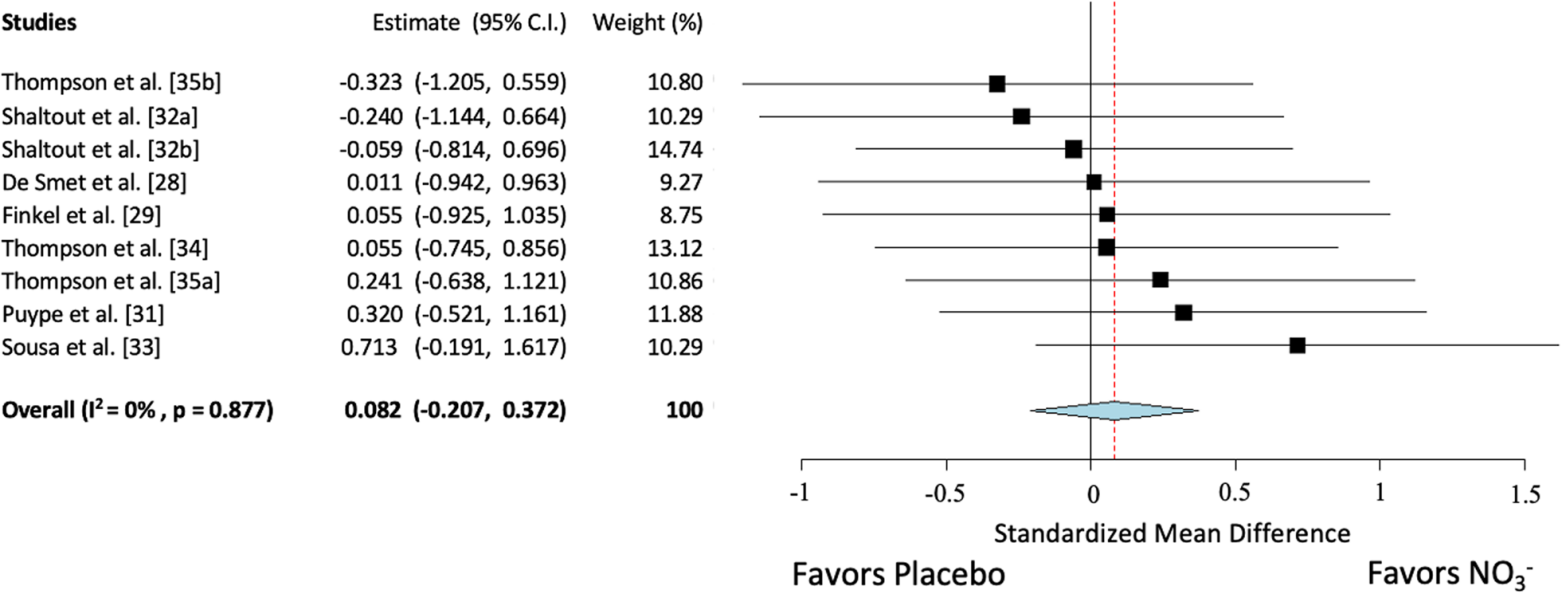
Forest Plot of Time to Exhaustion (TTE). [32a] Heart failure trial. [32b] Hypertension trial. [35a] Beetroot juice trial. [35b] Potassium nitrate trial

The overall model indicated that NO_3_^−^ supplementation did not improve exercise training responses on VO_2peak_ beyond exercise alone (n = 11 trials, SMD = 0.18; 95% confidence interval (CI): − 0.09, 0.44; *p* = 0.19; Fig. 2). There was no significant statistical heterogeneity present within this analysis (Q = 6.7, df = 10, *p* = 0.76, I^2^ = 0%, *p* = 0.76). Sensitivity analysis identified one trial [31] as potentially outlying/influential (Additional file 1: Figure S2), but removal of this trial had no significant effect on the observed effect. For the secondary outcome, the overall model indicated that NO_3_^−^ supplementation did not improve exercise training responses on TTE beyond exercise alone (n = 9 trials, SMD = 0.08; 95% CI: − 0.21, 0.37; *p* = 0.58, Fig. 3). There was no significant statistical heterogeneity present within this analysis (Q = 3.7; df = 8; *p* = 0.88; *I*^2^ = 0%, *p* = 0.88).

### Outliers, Influence, and Power

Examination of studentized residuals for trials included in the VO_2peak_ model were analyzed, revealing no indication of outliers in this model. One study was deemed influential according to Cook’s distance for VO_2peak_ [31]. Removal of this study did not change the lack of significant effect of NO_3_^−^ supplementation on training for the pooled outcome for VO_2peak_, although it did greatly reduce the effect size (SMD = 0.08; 95% CI: − 0.19, 0.36; *p* = 0.55). This study had large improvements in VO_2peak_ in the NO_3_^−^ group compared to the placebo group, but this study was identified as having “some concerns” in the risk of bias assessment due to the potential single-blinded nature of the study design. Visual inspection of the funnel plot for VO_2peak_ also revealed that the one study that was detected as influential by Cook’s distance also had a large effect size (Additional file 1: Figure S2), although neither the rank correlation nor the regression test indicated any funnel plot asymmetry (*p* = 0.76 and *p* = 0.96, respectively). All other studies removed during sensitivity analysis had no substantial effect on the overall model.

Examination of studentized residuals for studies included in the TTE model were analyzed, revealing no indication of outliers in this model. According to Cook’s distance, none of the studies were considered influential. Visual inspection of the funnel plot for TTE did not reveal any asymmetry (Additional file 1: Figure S3), and neither the rank correlation nor the regression test indicated any funnel plot asymmetry (*p* = 0.76 and *p* = 0.85, respectively). All studies removed during sensitivity analysis had no substantial effect on the overall model.

Examination of the sunset funnel plot (Additional file 1: Figure S4) shows that at the pre-determined α = 0.05, the median power of all trials included in this meta-analysis was 6.7%, with an average effect size of 0.68 and 1.07 required for statistical power levels of 33% and 66%, respectively.

### Subgroup Analysis

Moderator subgroup analyses for VO_2peak_ and TTE are shown in Tables 3 and 4, respectively. Subgroup analyses showed no significant moderation of sex, health status, route of NO_3_^−^ administration, level of training oxygenation, or the presence of an acute NO_3_^−^ dose prior to post-testing on either VO_2peak_ or TTE (all *p* > 0.05). Similarly, all meta-regressions performed did not reveal baseline VO_2peak_, age, body weight, duration of training intervention, NO_3_^−^ dose, or NO_3_^−^ dose normalized to bodyweight to be significant moderators on either VO_2peak_ or TTE (Table 5; all *p* > 0.05).

**Table 3:** Moderator subgroup analysis for VO_2peak_

Moderator variable	Comparisons		*p*-values
VO_2peak_ overall		SMD = 0.18 CI: − 0.09, 0.44	*p* = 0.19
Sex	Male:	SMD = 0.28 CI: − 0.21, 0.76	*p* = 0.27
	Mixed:	SMD = 0.10 CI: − 0.24, 0.44	*p* = 0.55
Health Status	Healthy:	SMD = 0.21 CI: − 0.11, 0.52	*p* = 0.20
	Clinical:	SMD = 0.11 CI: − 0.37, 0.58	*p* = 0.66
Oxygen	Normoxia:	SMD = 0.07 CI: − 0.23, 0.37	*p* = 0.65
	Hypoxia:	SMD = 0.48 CI: − 0.22, 1.19	*p* = 0.18
Route of Administration	BRJ:	SMD = 0.25 CI: − 0.05, 0.55	*p* = 0.10
	Other:	SMD = − 0.06 CI: − 0.59, 0.47	*p* = 0.83
Acute Dosing Prior	Yes	SMD = 0.23 CI: − 0.09, 0.55	*p* = 0.17
	No	SMD = 0.06 CI: − 0.39, 0.52	*p* = 0.78

*BRJ* beetroot juice, *CI* 95% Confidence interval, *SMD* Standardized mean difference

**Table 4:** Moderator subgroup analysis for TTE

Moderator variable	Comparisons		*p*-values
*TTE Overall*		SMD = 0.08 CI: − 0.21, 0.37	*p* = 0.58
Sex	Male:	SMD = 0.29 CI: − 0.17, 0.75	*p* = 0.21
	Mixed:	SMD = − 0.06 CI: − 0.43, 0.32	*p* = 0.76
Health Status	Healthy:	SMD = 0.15 CI: − 0.18, 0.49	*p* = 0.37
	Clinical:	SMD = − 0.13 CI: − 0.71, 0.45	*p* = 0.66
Oxygen	Normoxia:	SMD = − 0.04 CI: − 0.39, 0.31	*p* = 0.81
	Hypoxia:	SMD = 0.36 CI: − 0.16, 0.88	*p* = 0.18
Route of Administration	BRJ:	SMD = 0.14 CI: − 0.18, 0.46	*p* = 0.40
	Other:	SMD = − 0.15 CI: − 0.81, 0.50	*p* = 0.65
Acute Dosing Prior	Yes	SMD = 0.09 CI: − 0.23, 0.41	*p* = 0.57
	No	SMD = 0.03 CI: − 0.65, 0.72	*p* = 0.93

*BRJ* beetroot juice, *CI* 95% Confidence interval, *SMD* Standardized mean difference

**Table 5:** Meta-regression analysis for VO_2peak_ and TTE

Moderator Variable	VO_2peak_ Comparison	VO_2peak_ *p*-value	TTE Comparison	TTE *p*-value
Baseline VO_2peak_	ß = 0.01 CI: − 0.01, 0.02	0.48	ß = 0.01 CI: − 0.01, 0.03	0.25
Age	ß = − 0.003 CI: − 0.02, 0.01	0.70	ß = − 0.01 CI: − 0.02, 0.011	0.55
Weight	ß = − 0.02 CI: − 0.05, 0.01	0.27	ß = − 0.02 CI: − 0.05, 0.02	0.33
NO_3_^−^ mmol/day	ß = 0.02 CI: − 0.08, 0.11	0.74	ß = 0.01 CI: − 0.11, 0.12	0.91
NO_3_^−^ mmol/kg/day*	ß = − 2.24 CI: − 8.02, 3.53	0.45	ß = − 0.14 CI: − 6.68, 6.41	0.97
Training duration	ß = 0.04 CI: − 0.06, 0.15	0.42	ß = 0.01 CI: − 0.29, 0.31	0.94

*ß* Regression coefficient, *CI* 95% Confidence interval, *TTE* Time to Exhaustion

*Manually calculated based on mmol and mean kg data

### Sensitivity Analysis

As there is evidence in the literature suggesting that BRJ may provide more favorable benefits than other forms of NO_3_^−^ supplementation, a separate model was performed including only healthy participants who consumed NO_3_^−^ via BRJ (Additional file 1: Figure S5). Although this model revealed a trend for BRJ benefiting VO_2peak_ improvement beyond exercise training alone (SMD = 0.35; 95% CI: − 0.04, 0.75; *p* = 0.08), removal of the study previously identified as being influential [31] eliminated the observed trend (SMD = 0.16; 95% CI: − 0.01, 0.88; *p* = 0.46). Moderator analysis on this subset of data did not reveal any significant moderators (Additional file 1: Table S1; all *p* > 0.05). The sensitivity analysis model performed with TTE as an outcome showed no significant effect of NO_3_^−^ consumed as BRJ in healthy participants (SMD = 0.26; 95% CI: − 0.13, 0.65; *p* = 0.19) (Additional file 1: Figure S6). Moderator analysis on this subset did not reveal any significant moderators (Additional file 1: Table S2).

### GRADE Assessment

Using the GRADE Assessment, all outcomes ranged from “low” to “high” certainty of evidence (Additional file 1: Figure S7). VO_2peak_ for the overall pooled effects, as well as the sensitivity analysis for TTE in healthy participants consuming BRJ, were both downgraded to “moderate” certainty because of a potentially influential study and risk of publication bias. Sensitivity analysis on the outcome of VO_2peak_ in healthy participants consuming BRJ was downgraded to “low” because of both a potentially influential study and because two studies (of five total) with the largest effect were not described as double-blinded.

## Discussion

### Overall model

The use of inorganic NO_3_^−^ supplementation to improve physical function and exercise performance has increased over the last decade. To date, data demonstrating the benefits of acute and short-term NO_3_^−^ supplementation on exercise performance are equivocal, but the consensus is that NO_3_^−^ likely confers an overall small beneficial effect in individuals with low to moderate fitness. A summary of these data can be found in several meta-analyses and a recent expert consensus [15, 37–40]. Given that most athletes and clinical populations engaged in NO_3_^−^ supplementation are likely involved in chronic exercise training/rehabilitation to improve function/performance, it is important to examine the outcomes from exercise training and NO_3_^−^ supplementation in combination compared with training alone.

The results of this meta-analysis suggest that the addition of NO_3_^−^ supplementation to exercise training does not enhance VO_2peak_ or TTE beyond normal exercise training responses (Figs. 2 and 3, respectively), although it is important to note the limited number of studies and small sample sizes associated with studies in this area. As visualized in the sunset funnel plot (Additional file 1: Figure S4), existing studies are not sufficiently powered to detect the trivial-small effect observed in this meta-analysis. Further, while the present data suggest trivial improvements in VO_2peak_, these data are heavily influenced by a trial that was potentially single-blinded [31]. Removal of this study further weakened the effects of NO_3_^−^ supplementation. The limited existing data suggest that any improvement in VO_2peak_ and TTE observed after NO_3_^−^ supplementation were likely trivial, and that more studies with longer training durations and with larger sample sizes are needed.

### Subgroup Data

It has been hypothesized that NO_3_^−^ supplementation may produce greater benefits in hypoxic or low oxygen conditions [41], as well as in individuals with lower VO_2peak_ at baseline [42] who may have greater capacity to improve, in clinical populations with inhibited endogenous NO production via endothelial nitric oxide synthase (eNOS) [43], and in biological males (although this may be partly due to a lack of studies in females) [37]. Furthermore, evidence suggests that NO_3_^−^ may be most effective when delivered in more moderate doses (both absolute mmol and mmol/kg) [44–46], and when delivered in the form of beetroot juice rather than another form such as a nitrate salt [47, 48]. Accordingly, we sought *a priori* to determine the potential influence of these variables on the exercise training responses to NO_3_^−^ via a subgroup moderation. These results showed that there were no statistically significant moderators (Tables 2, 3, and 4).

### General Discussion

While these moderator variables have been proposed to predict acute exercise responses to NO_3_^−^ supplementation, it is possible that these acute changes are not large enough to result in an accumulated fitness benefit over time. Additionally, each moderator variable has considerations that may have impacted the lack of significant findings. For example, one moderator variable was baseline VO_2peak_, as those who are less aerobically fit initially have a lower VO_2peak_ and may be more likely to have limitations in their baseline endogenous production of NO [49, 50]. Theoretically, these individuals will have greater responses to training and to NO_3_^−^ supplementation, but this was not the case. For example, the study with the highest improvement in VO_2peak_ following NO_3_^−^ supplementation compared to control (∆ 5.5 vs. 3.0 ml/kg/min, respectively), was also the group with the highest baseline fitness (~ 60 mL/kg/min) [31], whereas the study group that experienced the largest difference in TTE following NO_3_^−^ supplementation compared to control (∆ -2 vs. -95 s, respectively), had the second highest baseline fitness (~ 56 mL/kg/min) [33]. Further, the VO_2peak_ improvements were low in the clinical populations, averaging only 0.55 ml/kg/min across all conditions, although this may be due to the more moderate exercise intensity used in these studies compared to the healthy population studies which all employed high intensity or sprint interval training [51].

While data have also suggested that moderate absolute doses of NO_3_^−^ have generally shown similar benefits to larger doses in exercise responses [45], there is increasing scientific interest in the effects of NO_3_^−^ dose normalized relative to body mass [44, 52]. Because of this, a moderator analysis was performed for dose of NO_3_^−^ expressed both as mmol/day as well as mmol/kg/day, but neither were shown to be significant predictors of outcomes (Table 5). Finally, biological sex has also been proposed as a factor impacting the beneficial effects of NO_3_^−^, suggesting a potential lack of impact in females compared to males [37, 53, 54], although there is a paucity of data examining the impact of NO_3_^−^ in females. Indeed, none of the studies involved in this meta-analysis assessed only females, and those that included both sexes [32, 34–36] did not explore sex-differences in responses. Whether NO_3_^–^ dose should be normalized to bodyweight has key implications in females due to the typically lower body mass compared to males and higher basal NO_2_^−^ levels [55]. As there appears to be a possible hormesis response to NO_3_^−^ supplementation in which an optimal dose outperforms a low or a high dose [46], it seems plausible that females are at risk of exceeding an optimal dose of NO_3_^−^ if given the same absolute dose shown to benefit males. Thus, the normalization of the dose of NO_3_^−^ to bodyweight, especially in females, merits further exploration.

Despite the lack of significant moderators, a trend was seen in improvement of VO_2peak_ in the BRJ subgroup (*p* = 0.10; Table 3). This was perhaps due to the presence of biologically active polyphenols, antioxidants, etc. that are found in BRJ [56] that may facilitate conversion of NO_2_^−^ to NO [57–60]. Indeed, BRJ has been shown to improve plasma NO_3_^−^, submaximal VO_2_, and TTE all to a greater extent than equimolar sodium nitrate [47, 48]. While not reaching statistical significance, this meta-analysis suggests that BRJ may offer greater physiological outcomes than other forms of NO_3_^−^ for improving the responses to exercise training. Further, trials included in this meta-analysis (all of which used BRJ) that included participants with clinical conditions had varying severity of disease (hypertension, peripheral arterial disease, and heart failure). These trials reported poor fitness improvements in the training interventions (NO_3_^−^: 0.75 ml/kg/min vs. placebo: 0.93 ml/kg/min average improvements; data not shown). While this was potentially due to the maladaptive and continually worsening pathology of some of these diseases, inclusion of these clinical populations in the meta-analysis may have weakened an overall training effect of the combined interventions. This prompted an additional sensitivity analysis in which a meta-analysis was performed on only studies using BRJ in healthy participants. This analysis revealed a stronger trend for improvement with BRJ supplementation in VO_2peak_ (*p* = 0.08; Additional file 1: Figure S5) compared with TTE (*p* = 0.19; Additional file 1: Figure S6). Further subgroup and meta-regression analysis revealed that there were no significant moderators (Additional file 1: Tables S1 and S2). While this subgroup involved only 5 trials (total n = 103), 1 of which was identified as being potentially influential (Fig. 2), this finding may suggest that NO_3_^−^ when administered in the form of BRJ could potentially improve exercise training responses to VO_2peak_ in healthy participants. While these data are underpowered as they stand, and any potential improvements in VO_2peak_ appear to be small, this suggests that additional research is needed to determine if BRJ supplementation may serve as an ergogenic aid in healthy individuals. While many collegiate and professional athletes are taking supplemental NO_3_^−^ (often via BRJ) in hopes of performance enhancement, existing data suggest these recommendations may be speculative and premature in nature.

Why chronic NO_3_^−^ supplementation does not appear to clearly improve exercise training benefits despite acute benefits on exercise is unknown. It appears plausible that improvements of each acute exercise bout throughout a training intervention would accumulate into a greater improvement in responses. VO_2peak_ is improved with exercise training by factors such as increases in stroke volume, arterio-venous oxygen extraction, and oxygen carrying capacity. Given the shorter duration of training in many of these studies, many training-induced improvements in VO_2peak_ are attributed to improvements in blood volume and the associated improvement in venous return and stroke volume [61, 62]. To our knowledge, there are no studies examining the effects of NO_3_^−^ supplementation on blood volume in humans. It is also possible that exercise training outcomes were not improved in this analysis because the adaptations induced by exercise training alone far exceed enhancements seen by simply supplementing with NO_3_^−^. Ultimately, more studies are needed to address these gaps in knowledge.

### Limitations

A major limitation of this meta-analysis is the low number of trials (n = 11) and the small sample sizes of trials included (average n = 20 per trial, 10 per group). The authors feel that this is important to point out as it highlights the need for more and larger studies addressing the ability of NO_3_^−^ supplementation to augment exercise training responses. This also applies to the subgroup analyses performed in this meta-analysis, as the subgroups will have less statistical power than the entire model. Of the 9 studies, 11 total trials were included in the meta-analysis as two of the studies had multiple study populations as well as a placebo group (BRJ and potassium nitrate (KNO3) [35]; heart failure and hypertension [32]). We recognize that including multiple trials from one study may contribute to analytical issues such as “double counting.” Because the placebo groups were the only groups with trials used twice, and these groups experienced expected training responses, this appears unlikely to have impacted the outcomes. Further, the NO_3_^−^ normalized to bodyweight data for each trial was calculated based on absolute values of NO_3_^−^ supplemented and mean bodyweight for the study. Although this is meant for exploratory purposes, caution should be warranted interpreting these data as they present risk of error due to the method of calculation. This meta-analysis was not pre-registered, although all outcomes and subgroup analyses were determined *a priori*.

Additionally, the inclusion criteria included a minimum of 3 weeks of exercise training with an average training time of 5 weeks (only 1 study beyond 6 weeks [36]). This relatively short duration is less likely to induce large changes in VO_2peak_ [63, 64] and thus may not be sufficient to provide an accurate representation of potential differences between treatments. On average, VO_2peak_ improved 4.0% and 6.4% for placebo and NO_3_^−^ supplemented groups after exercise training, respectively. It is possible that larger improvements as well as larger disparities between supplemented groups would be seen with longer supplementation and training time. Despite this, duration of training intervention was not a statistically significant moderator for any outcomes. The lack of additional exercise measures that are known to impact performance, such as lactate threshold, is another limitation to the results. However, large inconsistencies between studies (i.e., lactate samples taken during submaximal vs maximal workloads) did not allow for accurate analyses of these data. Additionally, NO_3_^−^ combined with exercise training may have an impact on measures of cardiovascular health which was not explored in this meta-analysis.

Finally, a limitation not of this meta-analysis, but rather of the trials themselves, is that the majority of the studies that observed exercise training with NO_3_^−^ supplementation also provided an acute dose of NO_3_^−^ prior to post-training testing [31–35]. This makes it impossible to determine whether any differences in training responses stemmed from the chronic training alongside NO_3_^−^, or whether the acute effects of NO_3_^−^ were simply the cause of any observed differences. Indeed, subgroup analysis showed this to be a significant moderator in the BRJ and healthy subset of data (Additional file 1: Table S1), and studies which included acute supplementation overall showed a general trend for greater improvements. Future studies in this field should abstain from an acute NO_3_^−^ dose prior to post-testing to determine a true training difference.

## Conclusions

The results of this systematic review and meta-analysis suggest that, based on the limited data available, NO_3_^−^ supplementation in addition to exercise training does not appear to improve VO_2peak_ or time to exhaustion above and beyond that of exercise training alone. Additionally, there were no statistically significant moderators observed (i.e., sex, health status, training oxygenation, route of NO_3_^−^ administration, baseline VO_2peak_, age, bodyweight, or NO_3_^−^ dose). A subset of studies revealed a trend for improvement in VO_2peak_ beyond exercise alone (*p* = 0.08) in healthy participants using BRJ as the mode of NO_3_^−^ administration, although these improvements were greatly impacted by a trial that was deemed influential and concerning in terms of bias. Ultimately, more studies with longer training duration, larger sample sizes, and the addition of examining sex as a biological variable are needed to determine whether NO_3_^−^ supplementation can improve exercise training responses, regarding VO_2peak_ and TTE, compared to exercise training alone. Because of this, caution is warranted for individuals supplementing with NO_3_^−^ in hopes of greater exercise training responses as the current data suggest trivial and non-significant improvements in these outcomes.

### Supplementary Information

**Additional file 1**: **Figure S1**: Risk of Bias Assessment. [32a] Heart failure trial. [32b] Hypertension trial. [35a] Beetroot juice trial. [35b] Potassium nitrate trial. **Figure S2**: Funnel Plot for VO_2peak_ as an outcome. **Figure S3**: Funnel Plot for Time to Exhaustion (TTE) as an outcome. **Figure S4**: The Sunset Funnel Plot. **Figure S5**: Sensitivity Analysis - Forest Plot of VO_2peak_ with inclusion only of studies which observed healthy participants and with beetroot juice supplementations as the administration route of NO_3_^−^. [32a] Heart failure trial. [32b] Hypertension trial. [35a] Beetroot juice trial. [35b] Potassium nitrate trial. **Table S1**: Subgroup Analysis for VO_2peak_ and Time to Exhaustion (TTE) in Studies Using Beetroot Juice (BRJ) in Healthy Subjects. **Figure S6**: Sensitivity Analysis - Forest Plot of Time to Exhaustion (TTE) with inclusion only of studies which observed healthy participants and with beetroot juice (BRJ) supplementations as the administration route of NO_3_^−^. [32a] Heart failure trial. [32b] Hypertension trial. [35a] BRJ trial. [35b] Potassium nitrate trial. **Table S2**: Meta-Regression Analysis for VO_2peak_ and Time to Exhaustion (TTE) in Studies Using Beetroot Juice (BRJ) in Healthy Subjects. **Figure S7**: GRADE Assessment of included trials.

## Data Availability

Available upon reasonable request.

## Abbreviations

VO_2peak_: Volume of oxygen uptake during peak exercise
NO_3_^−^: Nitrate
NO_2_^−^: Nitrite
NO: Nitric oxide
TTE: Time to exhaustion
SMD: Standardized mean difference
CI: Confidence interval
O_2_: Oxygen
PO_2_: Partial pressure of oxygen
a-vO_2_: Arterial-venous oxygen
RCT: Randomized controlled trial
kg: Kilogram
ß: Regression coefficient
RoB: Risk of bias
eNOS: Endothelial nitric oxide synthase
KNO_3_: Potassium nitrate
BRJ: Beetroot juice
