# Supplementary material for: Limited Effects of Inorganic Nitrate Supplementation on Exercise Training Responses: A Systematic Review and Meta-analysis
Source: Sports Med Open. 2023 Sep 11;9:84. doi: 10.1186/s40798-023-00632-1 (PMC10495291; doi:10.1186/s40798-023-00632-1)
Supplement: Supplementary file 1 — Additional file 1: Figure S1: Risk of Bias Assessment. [32a] Heart failure trial. [32b] Hypertension trial. [35a] Beetroot juice trial. [35b] Potassium nitrate trial. Figure S2: Funnel Plot for VO2peak as an outcome. Figure S3: Funnel Plot for Time to Exhaustion (TTE) as an outcome. Figure S4: The Sunset Funnel Plot. Figure S5: Sensitivity Analysis - Forest Plot of VO2peak with inclusion only of studies which observed healthy participants and with beetroot juice supplementations as the administration route of NO3−. [32a] Heart failure trial. [32b] Hypertension trial. [35a] Beetroot juice trial. [35b] Potassium nitrate trial. Table S1: Subgroup Analysis for VO2peak and Time to Exhaustion (TTE) in Studies Using Beetroot Juice (BRJ) in Healthy Subjects. Figure S6: Sensitivity Analysis - Forest Plot of Time to Exhaustion (TTE) with inclusion only of studies which observed healthy participants and with beetroot juice (BRJ) supplementations as the administration route of NO3−. [32a] Heart failure trial. [32b] Hypertension trial. [35a] BRJ trial. [35b] Potassium nitrate trial. Table S2: Meta-Regression Analysis for VO2peak and Time to Exhaustion (TTE) in Studies Using Beetroot Juice (BRJ) in Healthy Subjects. Figure S7: GRADE Assessment of included trials. [file 40798_2023_632_MOESM1_ESM.docx]

**
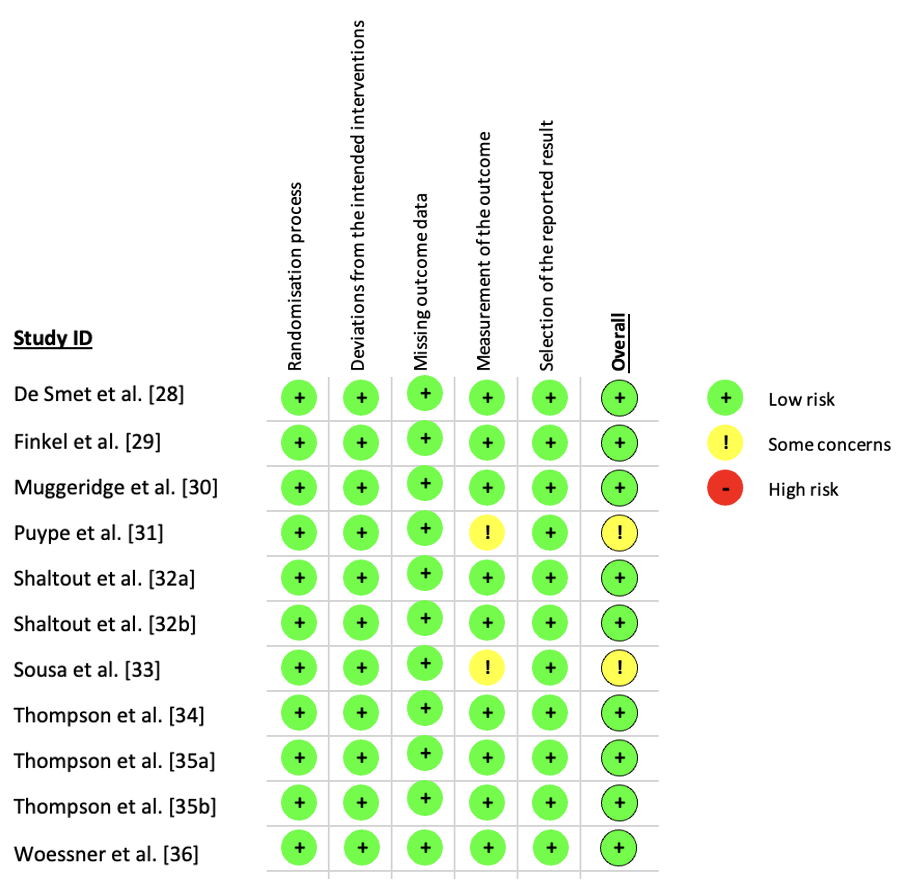
**

Figure S1: Risk of Bias Assessment.


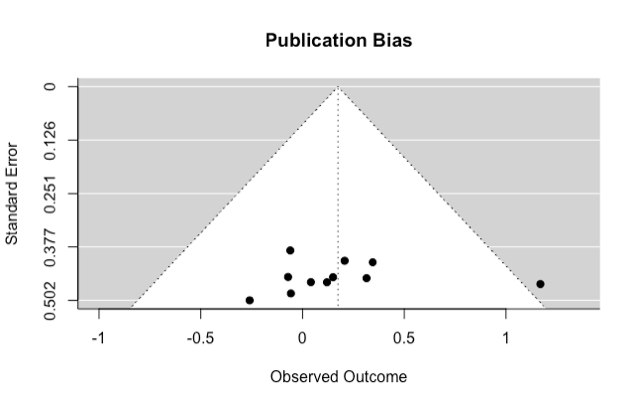


Figure S2: Funnel Plot for VO_2peak_ as an outcome.


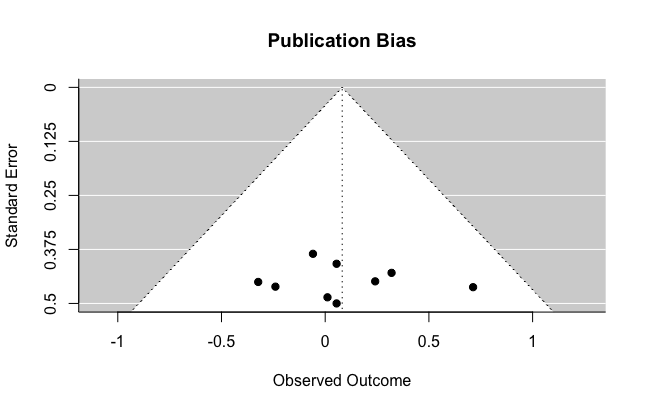


Figure S3: Funnel Plot for Time to Exhaustion (TTE) as an outcome.

Figure S4: The Sunset Funnel Plot.


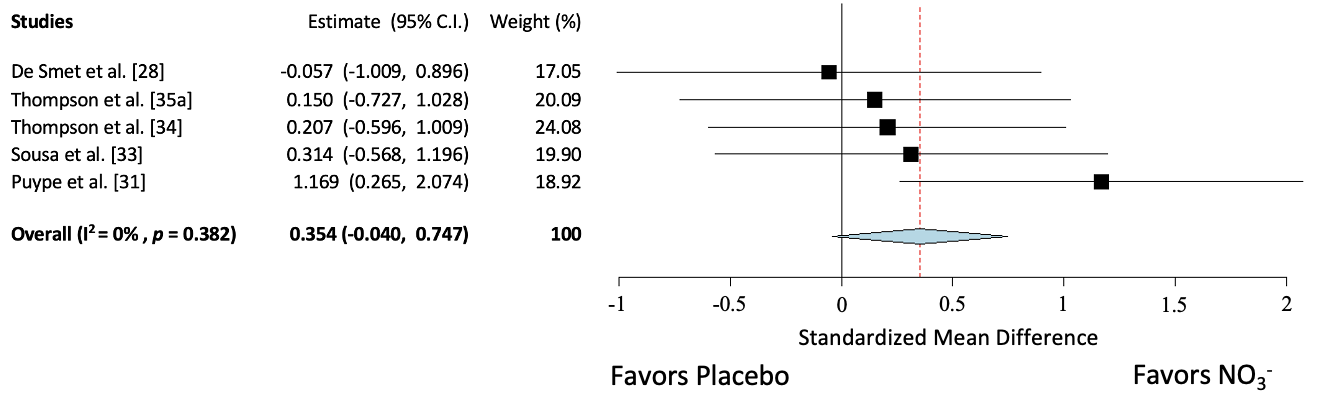


Figure S5: Sensitivity Analysis - Forest Plot of VO_2peak_ with inclusion only of studies which observed healthy participants and with beetroot juice supplementations as the administration route of NO_3_^-^ .

| **Table S1: Subgroup Analysis for VO_2peak_ and TTE in Studies Using BRJ in Healthy Subjects** | | | |
| --- | --- | --- | --- |
| **Moderator Variable** | **Comparisons** | | ***p*-values** |
| ***VO_2peak_ Overall*** | | SMD = 0.35  CI: -0.04, 0.75 | *p* = 0.08 |
| Sex | Male: | SMD = 0.48  CI: -0.22, 1.19 | *p* = 0.18 |
|  | Mixed: | SMD = 0.18  CI: -0.41, 0.77 | *p* = 0.55 |
| Oxygen | Normoxia: | SMD = 0.48  CI: -0.22, 1.19 | *p* = 0.18 |
|  | Hypoxia: | SMD = 0.18  CI: -0.41, 0.77 | *p* = 0.55 |
| Acute Dosing Prior | Yes | SMD = 0.44  CI: -0.01, 0.88 | *p* = 0.05 |
|  | No | SMD = -0.06  CI: -1.01, 0.90 | *NA* |
| ***Time to Exhaustion Overall*** | | SMD = 0.26  CI: -0.13, 0.65 | *p* = 0.19 |
| Sex | Male: | SMD = 0.36  CI: -0.16, 0.88 | *p* = 0.18 |
|  | Mixed: | SMD = 0.14  CI: -0.45, 0.73 | *p* = 0.65 |
| Oxygen | Normoxia: | SMD = 0.36  CI: -0.16, 0.88 | *p* = 0.16 |
|  | Hypoxia: | SMD = 0.14  CI: -0.45, 0.73 | *p* = 0.64 |
| Acute Dosing Prior | Yes | SMD = 0.31  CI: -0.11, 0.74 | *p* = 0.15 |
|  | No | SMD = 0.01  CI: -0.94, 0.96 | *NA* |
| BRJ: Beetroot juice. CI: 95% Confidence interval. SMD: Standardized mean difference. TTE: Time to Exhaustion. Note: *p*-value = NA due to single study inclusion in subgroup analysis. | | | |


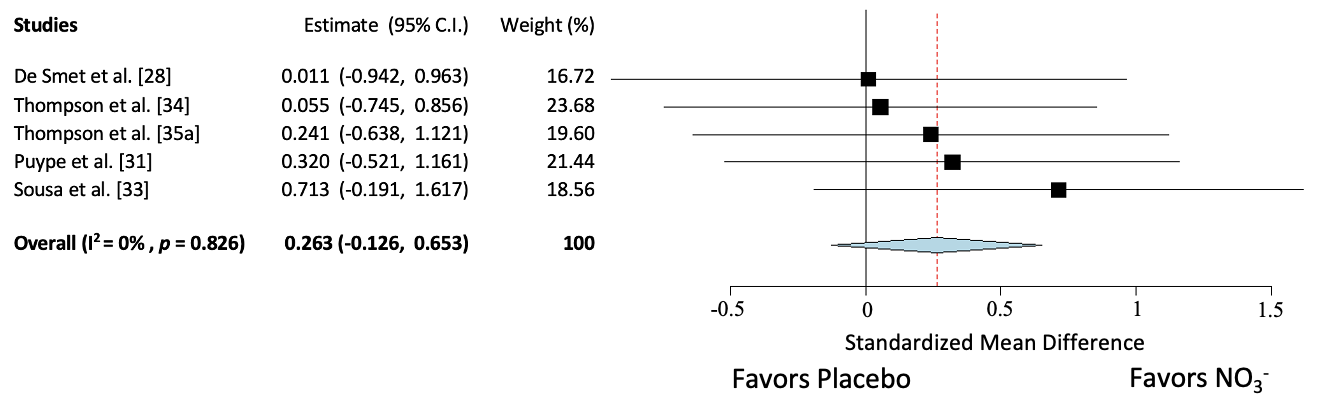


Figure S6: Sensitivity Analysis - Forest Plot of TTE with inclusion only of studies which observed healthy participants and with beetroot juice supplementations as the administration route of NO_3_^-^

| **Table S2: Meta-Regression Analysis for VO_2peak_ and TTE in Studies Using BRJ in Healthy Subjects** | | | | | |
| --- | --- | --- | --- | --- | --- |
| **Moderator Variable** | **VO_2peak_**  **Comparison** | **VO_2peak_**  ***p*-value** |  | **TTE**  **Comparison** | **TTE**  ***p*-value** |
| Baseline VO_2peak_ | ß = 0.04  CI: -0.02, 0.10 | 0.17 |  | ß = 0.02  CI: -0.04, 0.08 | 0.55 |
| Age | ß = -0.02  CI: -0.09, 0.06 | 0.63 |  | ß = -0.04  CI: -0.04, 0.11 | 0.35 |
| Weight | ß = -0.08  CI: -0.21, 0.06 | 0.28 |  | ß = -0.06  CI: -0.19, 0.08 | 0.42 |
| NO_3_^-^  mmol/day | ß = 0.04  CI: -0.12, 0.20 | 0.62 |  | ß = 0.02  CI: -0.18, 0.14 | 0.81 |
| NO_3_^-^ mmol/kg/day* | ß = -4.58  CI: -13.34, 4.19 | 0.31 |  | ß = -1.05  CI: -9.61, 7.51 | 0.81 |
| Training Duration | ß = 0.38  CI: -0.12, 0.88 | 0.13 |  | ß = -0.03  CI: -0.50, 0.50 | 0.91 |
| BRJ: Beetroot juice. TTE: Time to Exhaustion. ß: Regression coefficient. CI: 95% Confidence interval. *: manually calculated based on mmol and mean kg data. | | | | | |


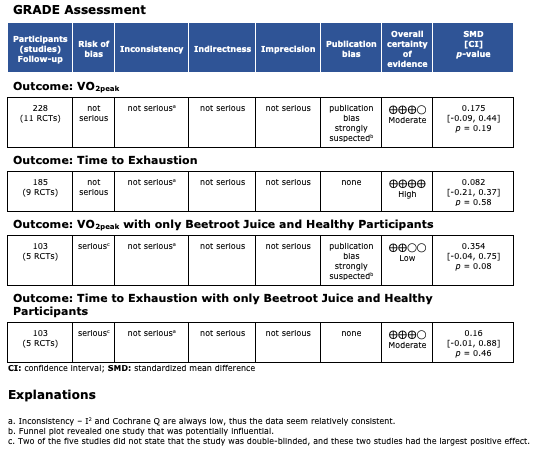


Figure S7: GRADE Assessment of included trials.
